# Supplementary material for: Hot spot prediction in protein-protein interactions by an ensemble system
Source: BMC Syst Biol. 2018 Dec 31;12(Suppl 9):132. doi: 10.1186/s12918-018-0665-8 (PMC6311905; doi:10.1186/s12918-018-0665-8)
Supplement: Supplementary file 2 — All relevant MATLAB code. A simple Matlab implement of our predictor. (ZIP 19,742 kb) [file 12918_2018_665_MOESM2_ESM.zip › Additional file 2/Read ASA/ASA website/SKEMPI/1H9DB.htm]

 NetSurfP - Protein Surface Accessibility and Secondary Structure Predictions

  

|  |  |  |
| --- | --- | --- |
|  |  | NetSurfP - Protein Surface Accessibility and Secondary Structure Predictions  Technical University of Denmark |

  


---

```
# For publication of results, please cite:
# A generic method for assignment of reliability scores applied to solvent accessibility predictions.
# Bent Petersen, Thomas Nordahl Petersen, Pernille Andersen, Morten Nielsen and Claus Lundegaard
# BMC Structural Biology 2009, 9:51 doi:10.1186/1472-6807-9-51
#
# Column 1: Class assignment - B for buried or E for Exposed - Threshold: 25% exposure, but not based on RSA
# Column 2: Amino acid
# Column 3: Sequence name
# Column 4: Amino acid number
# Column 5: Relative Surface Accessibility - RSA
# Column 6: Absolute Surface Accessibility
# Column 7: Z-fit score for RSA prediction
# Column 8: Probability for Alpha-Helix
# Column 9: Probability for Beta-strand
# Column 10: Probability for Coil
E P  1H9DB                  1    0.738 104.665  -0.845   0.003   0.003   0.994
E R  1H9DB                  2    0.596 136.392  -0.423   0.004   0.138   0.858
E V  1H9DB                  3    0.456  70.133  -0.775   0.019   0.141   0.840
E V  1H9DB                  4    0.260  39.885   0.501   0.018   0.088   0.893
E P  1H9DB                  5    0.577  81.919  -0.417   0.113   0.043   0.844
E D  1H9DB                  6    0.585  84.270  -0.470   0.257   0.016   0.727
E Q  1H9DB                  7    0.395  70.583  -0.312   0.257   0.016   0.727
E R  1H9DB                  8    0.448 102.638   0.122   0.257   0.016   0.727
E S  1H9DB                  9    0.423  49.564  -0.460   0.184   0.043   0.773
E K  1H9DB                 10    0.533 109.618   0.886   0.191   0.086   0.723
B F  1H9DB                 11    0.138  27.636   0.069   0.113   0.087   0.800
E E  1H9DB                 12    0.521  91.036  -0.244   0.113   0.043   0.844
E N  1H9DB                 13    0.401  58.692  -0.725   0.257   0.016   0.727
E E  1H9DB                 14    0.453  79.192  -0.222   0.858   0.002   0.139
E E  1H9DB                 15    0.586 102.357   0.194   0.923   0.002   0.076
B F  1H9DB                 16    0.137  27.456   0.261   0.923   0.002   0.076
B F  1H9DB                 17    0.049   9.854   0.316   0.938   0.007   0.055
E R  1H9DB                 18    0.398  91.028   1.422   0.879   0.010   0.111
E K  1H9DB                 19    0.403  82.959   1.150   0.802   0.014   0.185
B L  1H9DB                 20    0.063  11.499  -0.102   0.622   0.015   0.363
B S  1H9DB                 21    0.241  28.233  -1.127   0.257   0.016   0.727
E R  1H9DB                 22    0.521 119.263  -0.355   0.115   0.016   0.868
E E  1H9DB                 23    0.480  83.926  -0.747   0.113   0.043   0.844
B C  1H9DB                 24    0.202  28.305  -0.887   0.118   0.150   0.732
E E  1H9DB                 25    0.331  57.861  -0.441   0.066   0.296   0.638
B I  1H9DB                 26    0.126  23.273  -0.249   0.074   0.484   0.442
E K  1H9DB                 27    0.428  88.101   0.573   0.079   0.592   0.329
B Y  1H9DB                 28    0.190  40.496  -0.145   0.074   0.484   0.442
E T  1H9DB                 29    0.321  44.495  -0.622   0.066   0.296   0.638
B G  1H9DB                 30    0.291  22.941  -1.632   0.019   0.141   0.840
B F  1H9DB                 31    0.194  39.036   0.216   0.018   0.088   0.893
E R  1H9DB                 32    0.540 123.729  -0.001   0.018   0.047   0.935
E D  1H9DB                 33    0.572  82.382  -1.767   0.018   0.019   0.964
E R  1H9DB                 34    0.360  82.509  -0.230   0.018   0.019   0.964
E P  1H9DB                 35    0.382  54.192  -0.163   0.058   0.017   0.925
E H  1H9DB                 36    0.401  72.924  -0.152   0.600   0.003   0.397
E E  1H9DB                 37    0.544  95.107  -0.543   0.858   0.002   0.139
E E  1H9DB                 38    0.484  84.467   0.120   0.858   0.002   0.139
B R  1H9DB                 39    0.204  46.624  -0.287   0.923   0.002   0.076
B Q  1H9DB                 40    0.247  44.150  -0.148   0.923   0.002   0.076
E A  1H9DB                 41    0.425  46.791  -0.176   0.970   0.001   0.030
E R  1H9DB                 42    0.342  78.204   0.869   0.970   0.001   0.030
B F  1H9DB                 43    0.068  13.547  -0.521   0.923   0.002   0.076
E Q  1H9DB                 44    0.305  54.455   0.828   0.923   0.002   0.076
E N  1H9DB                 45    0.467  68.398  -0.181   0.858   0.002   0.139
B A  1H9DB                 46    0.295  32.542   0.250   0.694   0.003   0.303
B C  1H9DB                 47    0.055   7.694   0.116   0.622   0.015   0.363
E R  1H9DB                 48    0.468 107.172   0.403   0.561   0.047   0.393
E D  1H9DB                 49    0.619  89.169  -0.430   0.181   0.016   0.803
E G  1H9DB                 50    0.532  41.876  -0.985   0.058   0.017   0.925
E R  1H9DB                 51    0.408  93.340  -0.271   0.053   0.043   0.903
B S  1H9DB                 52    0.159  18.600  -0.228   0.056   0.142   0.802
E E  1H9DB                 53    0.380  66.369   0.452   0.074   0.484   0.442
B I  1H9DB                 54    0.061  11.340   0.435   0.021   0.756   0.223
B A  1H9DB                 55    0.158  17.445   0.058   0.018   0.846   0.136
B F  1H9DB                 56    0.103  20.592  -0.021   0.018   0.846   0.136
B V  1H9DB                 57    0.089  13.710  -0.247   0.018   0.846   0.136
B A  1H9DB                 58    0.109  12.001  -1.265   0.021   0.756   0.223
B T  1H9DB                 59    0.216  29.931  -0.852   0.022   0.552   0.426
B G  1H9DB                 60    0.103   8.114  -1.833   0.022   0.359   0.619
B T  1H9DB                 61    0.191  26.464  -0.817   0.022   0.359   0.619
B N  1H9DB                 62    0.237  34.653  -1.492   0.022   0.359   0.619
B L  1H9DB                 63    0.098  17.980  -0.872   0.021   0.451   0.528
E S  1H9DB                 64    0.313  36.648  -0.393   0.022   0.552   0.426
B L  1H9DB                 65    0.131  24.078  -0.971   0.023   0.655   0.322
B Q  1H9DB                 66    0.253  45.168  -0.609   0.023   0.655   0.322
B F  1H9DB                 67    0.130  26.031  -1.770   0.022   0.552   0.426
B F  1H9DB                 68    0.209  41.946   0.052   0.004   0.197   0.799
B P  1H9DB                 69    0.261  37.064  -1.881   0.018   0.088   0.893
E T  1H9DB                 70    0.316  43.774  -0.808   0.018   0.019   0.964
E P  1H9DB                 71    0.469  66.551  -0.970   0.018   0.019   0.964
E S  1H9DB                 72    0.502  58.799  -1.297   0.018   0.019   0.964
E R  1H9DB                 73    0.589 134.904  -0.368   0.053   0.043   0.903
E E  1H9DB                 74    0.552  96.365   0.151   0.056   0.142   0.802
E Y  1H9DB                 75    0.364  77.723  -0.207   0.066   0.296   0.638
B V  1H9DB                 76    0.110  16.846   0.011   0.069   0.386   0.545
E D  1H9DB                 77    0.401  57.726   0.236   0.069   0.386   0.545
B L  1H9DB                 78    0.151  27.557   0.296   0.064   0.216   0.721
E E  1H9DB                 79    0.568  99.265  -0.456   0.056   0.142   0.802
E R  1H9DB                 80    0.518 118.714  -0.064   0.056   0.142   0.802
E E  1H9DB                 81    0.540  94.251  -0.667   0.020   0.205   0.775
E A  1H9DB                 82    0.468  51.596  -0.311   0.020   0.205   0.775
B G  1H9DB                 83    0.285  22.422  -0.768   0.004   0.420   0.576
E K  1H9DB                 84    0.383  78.865   1.247   0.002   0.816   0.182
B V  1H9DB                 85    0.057   8.699   0.450   0.001   0.900   0.099
E Y  1H9DB                 86    0.248  52.912   0.641   0.001   0.959   0.040
B L  1H9DB                 87    0.058  10.601   0.210   0.001   0.900   0.099
E K  1H9DB                 88    0.371  76.376   0.244   0.002   0.816   0.182
B A  1H9DB                 89    0.079   8.673  -1.384   0.005   0.336   0.660
E P  1H9DB                 90    0.345  48.927  -0.135   0.020   0.205   0.775
B M  1H9DB                 91    0.063  12.686   0.197   0.004   0.420   0.576
B I  1H9DB                 92    0.072  13.320  -0.366   0.004   0.616   0.381
B L  1H9DB                 93    0.060  11.023  -0.843   0.004   0.514   0.481
B N  1H9DB                 94    0.183  26.777  -1.106   0.021   0.279   0.699
B G  1H9DB                 95    0.072   5.690  -1.472   0.004   0.420   0.576
B V  1H9DB                 96    0.046   7.132  -0.173   0.001   0.900   0.099
B C  1H9DB                 97    0.029   4.001   0.782   0.006   0.962   0.032
B V  1H9DB                 98    0.022   3.335   1.312   0.006   0.962   0.032
B I  1H9DB                 99    0.073  13.450   0.590   0.006   0.962   0.032
B W  1H9DB                100    0.056  13.468   0.059   0.001   0.900   0.099
B K  1H9DB                101    0.206  42.354   0.636   0.002   0.816   0.182
B G  1H9DB                102    0.089   6.989  -1.939   0.023   0.655   0.322
B W  1H9DB                103    0.129  31.000  -0.104   0.021   0.756   0.223
B I  1H9DB                104    0.084  15.485  -0.379   0.021   0.756   0.223
B D  1H9DB                105    0.219  31.500  -0.372   0.021   0.451   0.528
B L  1H9DB                106    0.146  26.788  -0.124   0.066   0.296   0.638
E Q  1H9DB                107    0.414  73.958  -0.282   0.069   0.386   0.545
E R  1H9DB                108    0.377  86.402   0.147   0.066   0.296   0.638
B L  1H9DB                109    0.109  19.995  -0.117   0.066   0.296   0.638
E D  1H9DB                110    0.496  71.502  -0.623   0.019   0.141   0.840
E G  1H9DB                111    0.375  29.528  -2.271   0.019   0.141   0.840
B M  1H9DB                112    0.279  55.748  -0.796   0.021   0.279   0.699
B G  1H9DB                113    0.220  17.298  -1.280   0.069   0.386   0.545
B C  1H9DB                114    0.202  28.347   0.099   0.079   0.592   0.329
B L  1H9DB                115    0.118  21.679   0.689   0.079   0.592   0.329
E E  1H9DB                116    0.414  72.273   0.075   0.159   0.516   0.325
B F  1H9DB                117    0.191  38.253   0.433   0.352   0.332   0.316
E D  1H9DB                118    0.332  47.899   0.307   0.455   0.046   0.498
E E  1H9DB                119    0.555  96.906  -0.091   0.802   0.014   0.185
E E  1H9DB                120    0.593 103.562   0.249   0.831   0.044   0.125
E R  1H9DB                121    0.394  90.180   0.474   0.802   0.014   0.185
E A  1H9DB                122    0.307  33.842  -0.929   0.717   0.014   0.269
E Q  1H9DB                123    0.657 117.269   0.219   0.522   0.016   0.462
E Q  1H9DB                124    0.684 122.145   0.208   0.339   0.016   0.645
E E  1H9DB                125    0.892 155.780   0.503   0.003   0.003   0.994

Explain the output.  Go back.
```

---
